# COVID-19 control in low-income settings and displaced populations: what can realistically be done?

**DOI:** 10.1186/s13031-020-00296-8

**Published:** 2020-07-31

**Authors:** Maysoon Dahab, Kevin van Zandvoort, Stefan Flasche, Abdihamid Warsame, Ruwan Ratnayake, Caroline Favas, Paul B. Spiegel, Ronald J. Waldman, Francesco Checchi

**Affiliations:** 1 Conflict & Health Research Group, King’s Centre for Global Health and Health Partnerships, King’s College London, London, UK; 2 Department of Infectious Disease Epidemiology, Faculty of Epidemiology and Population Health, London School of Hygiene and Tropical Medicine, London, UK; 3 Centre for Humanitarian Health, Johns Hopkins University Bloomberg School of Public Health, Baltimore, MD USA; 4 Department of Global Health, Milken Institute School of Public Health, George Washington University, Washington, DC USA; 5 Doctors of the World USA, New York, NY USA

## Abstract

COVID-19 prevention strategies in resource limited settings, modelled on the earlier response in high income countries, have thus far focused on draconian containment strategies, which impose movement restrictions on a wide scale. These restrictions are unlikely to prevent cases from surging well beyond existing hospitalisation capacity; not withstanding their likely severe social and economic costs in the long term.

We suggest that in low-income countries, time limited movement restrictions should be considered primarily as an opportunity to develop sustainable and resource appropriate mitigation strategies. These mitigation strategies, if focused on reducing COVID-19 transmission through a triad of prevention activities, have the potential to mitigate bed demand and mortality by a considerable extent. This triade is based on a combination of high-uptake of community led shielding of high-risk individuals, self-isolation of mild to moderately symptomatic cases, and moderate physical distancing in the community.

We outline a set of principles for communities to consider how to support the protection of the most vulnerable, by shielding them from infection within and outside their homes. We further suggest three potential shielding options, with their likely applicability to different settings, for communities to consider and that would enable them to provide access to transmission-shielded arrangements for the highest risk community members. Importantly, any shielding strategy would need to be predicated on sound, locally informed behavioural science and monitored for effectiveness and evaluating its potential under realistic modelling assumptions. Perhaps, most importantly, it is essential that these strategies not be perceived as oppressive measures and be community led in their design and implementation. This is in order that they can be sustained for an extended period of time, until COVID-19 can be controlled or vaccine and treatment options become available.

## Background

Modelling predictions [1] suggest that uncontrolled COVID-19 epidemics will result in 7.0 billion infections and 40 million deaths globally this year, with the impact expected to be most severe in low-income settings and forcibly displaced populations [2]. Three mechanisms could determine this: (i) higher transmissibility due to larger household sizes [3], intense social mixing [4] between the young and elderly [5], overcrowding in urban slums and displaced people’s camps, inadequate water and sanitation, and specific cultural and faith practices such as mass prayer gatherings, large weddings and funerals during which super-spreading events might propagate transmission disproportionately [6]; (ii) higher infection-to-case ratios and progression to severe disease due to the virus’ interaction with highly prevalent co-morbidities, including non-communicable diseases (NCDs; prevalence of hypertension and diabetes is often higher in low- than high-income settings [7, 8], with a far lower treatment coverage [9]), undernutrition, tuberculosis [10, 11] and HIV^10^; and (iii) higher case-fatality due to a dire lack of intensive care capacity, especially outside large cities. Moreover, extreme pressure on curative health services could result in indirect impacts resulting from disrupted care for health problems other than COVID-19 [12]. These risks are exacerbated by the inadequacy of pandemic plans that enable public health systems to respond [13] adequately and without delay. While it remains to be seen if these risk factors can be counterbalanced by younger age distributions, on balance we believe that, given current evidence and plausible reasoning, drastic action is required immediately to protect the world’s most fragile populations from this unfolding threat. We present here our views on the challenges of current containment focused approaches contrasted to more realistic, economically and socially amenable interventions, to limit COVID-19 transmission even in the most resource-constrained settings.

## Containment may buy some time – at best

In recent months low-income and crisis-affected countries have followed a global pattern of attempting to interrupt further importation of COVID-19 from abroad through border closures. Examples from China, South Korea and Singapore [14] suggest that this approach may enable containment at least for some time; it is, however, very resource-intensive, and will inevitably require widespread testing and meticulous contact tracing [15]. It is doubtful that these measures are replicable in low-income and crisis settings, where inadequate surveillance and less-than-sufficient testing may initially obfuscate the true extent of locally driven transmission.

Current mathematical modelling forecasts [16] predict that even stringent lockdowns, similar to those implemented in Europe, would be unlikely to prevent cases from surging well beyond these countries’ existing hospitalisation capacity. Despite this, low-income countries are increasingly adopting population wide physical distancing strategies, where all residents except essential workers are asked to stay at home. This presents a difficult balance for low-income countries where the social and economic cost of population-wide physical distancing and travel restrictions, if sustained over a long period, could be very harmful for fragile, export-dependent economies and stretch livelihoods beyond people’s coping ability, in turn dis-incentivising adherence to control measures. In crises, both a lack of trust between populations and national authorities and on-going conflict can undermine a sweeping, one-size-fits-all strategy [17], especially if applied coercively. In short, a draconian containment strategy may be useful for a limited time to allow countries to better prepare, but risks failing beyond a horizon of weeks.

## What can realistically be done?

Of the three mechanisms we describe above, two (higher infection severity and case-fatality) appear less tractable for the time being. Some interventions to mitigate secondary impacts could help and should be pursued quickly (e.g. avoiding treatment interruptions for NCDs, TB and HIV by supplying patients with several months of medication through community health workers or dedicated clinics that can reach these patients; intermittent presumptive treatment to reduce other co-morbidities; freeing up health care capacity by postponing non-essential services). Options for improving oxygen therapy in low-resource settings could be explored [18]. However, there appears to be little realistic prospect of scaling up intensive care to the levels required for COVID-19, where demand in a typical low-income setting for critical care beds, even in the mitigated scenarios, is predicted to outstrip supply by a factor of 25 [19]. Isolation of cases in dedicated, but not high-intensity wards might offer neither clinical benefit nor meaningful transmission reductions, as most transmission would still be attributable [20] to low-risk infections [21] and the proportion of the infectiousness period spent pre-admission [22, 23], e.g. among household members. Moreover, without sufficient training and infection control supplies, such facilities would pose a major threat to the health of clinicians, already a very scarce resource in most low-income and crisis settings. However for the majority of LICs, where testing is not widely available, syndromic diagnosis could also result in inadvertent mixing and thus cross-transmission between true COVID-19 cases and patients with other acute respiratory infections.

By contrast, the mechanism of higher transmissibility appears more amenable to economically and socially feasible interventions, even in the most resource-constrained settings. Here too, however, a range of possible strategies may be considered. In order for population-wide physical distancing measures, increasingly being pursued in low-income settings, to realistically achieve sufficient impact on their own, these would require most non-essential workers to work from home or not at all, a strategy ill-suited to the economies and remote-working capability of low-income settings. Moreover, this must be sustained over a long period, until a vaccine, treatment or both are available at scale. We thus suggest that, where dispersive strategies targeting the general population are difficult to implement, enforce and/or sustain [24–26], leading to ongoing transmission among low-risk populations [27, 28], it will be crucial to focus resources on maximizing the impact of strategies to protect those most vulnerable to severe outcomes, in essence shielding them to the extent possible from the risk of infection. A recent modelling study [16] of the epidemic in three African countries (representing the range of age distributions on the continent) suggests that a combination of self-isolation, moderate distancing and high-uptake shielding would mitigate bed demand and mortality to a considerable extent.

## Shielding high-risk populations: general principles

In Ebola epidemics, isolating the ill into a contaminated ‘red zone’ is mainly needed to protect the healthy. For COVID-19, this paradigm is upturned: from the perspective of the highest risk groups, unless they can be shielded from infection and cared for while shielding, the red zone is everywhere. While stressing that no single approach is likely to fit all low-income or crisis settings, we outline below a set of principles that, implemented together, could support the general aim of protecting those most vulnerable from infection by helping them to live safely, dignifiedly and separately from their families and neighbours for what could be an extended period of time, until COVID-19 can be controlled or vaccine and treatment options become available.

### Who should be shielded?

Evolving information so far suggests a rapid increase in COVID-19 severity and mortality with age with a particularly high risk among people aged above 70 years and/or living with NCDs and other immuno-suppressing conditions [29, 30]. In the absence of evidence to the contrary we suggest that in low-income or crisis-affected populations the high-risk definition could be extended to those aged 60 years or above (a more meaningful proxy of old age in countries that have not completed the epidemiologic and demographic transition). It should also expressly consider those living with TB or HIV, and malnourished adults. TB patients, however, would likely need dedicated isolation arrangements in order to avoid close-quarters TB transmission.

### How should effective shielding be achieved?

Table 1 suggests three options for housing high-risk community members into transmission-shielded arrangements, with their likely applicability to different settings. Under options 1 and 2, it may be assumed that healthy household members are able to care (e.g. bathe, feed) for those with disabilities and the elderly; including low-risk carers (particularly those previously infected and thus probably immune) could also be an option. In the majority of communities with single room dwellings where option 1 is not possible, option 2 may be a viable option. While an extreme version of option 3, namely resettling large numbers (e.g. many hundreds) of high-risk people in dedicated buildings or neighbourhoods, might also be conceivable, we have discounted it due to likely high cost, potential lack of acceptance of elders being separated from families, and the risk of large-scale harm if transmission is seeded within such a concentrated ‘green zone’.

**Table 1:** Options for housing high-risk persons into designated ‘green zones’

Option	Description	Applicability	Notes
1. Household-level shielding	Each household demarcates a room or shelter for high-risk members. If necessary, a carer from the household is isolated with them.	Settings with multi-shelter compounds or multi-room houses.	Likely preferable to families with space available but also more likely to be ‘leaky’ if isolation is not strictly enforced.
2. Street- or extended family-level shielding	Neighbouring households (e.g. 5–10) or members of an extended family within a defined geographic locale (neighbourhood, district) voluntarily ‘house-swap’ and group their high-risk members into dedicated houses / shelters.	All, but especially urban settings.	Infection control and physical distancing measures would also have to be strictly observed within each green zone.
3. Neighbourhood- or sector-level isolation	Sections of the settlement are put aside for groups of high-risk people (e.g. 50–100).	High density camps/settlements (e.g. refugee/IDP camps, slums, informal settlements for migrants where humanitarian actors can provide supportive services and smaller scale isolation is not possible.	Ideally located at the periphery of camps to facilitate such measures. Infection control and physical distancing measures would also have to be strictly observed within each green zone. We have discounted this option due to likely high cost, potential lack of acceptance of elders being separated from families, and the risk of large-scale harm if transmission is seeded within such a concentrated ‘green zone’.

### Social mobilization, community engagement, and coordination

As demonstrated globally, risk communication of the urgency of an unseen threat is difficult. It is essential that these strategies not be perceived as an oppressive measure and have strong buy-in from the communities. All potential shielding strategies must be discussed from the beginning with communities so that they may be able to spontaneously and rapidly self-organize along not only epidemiologically sound principles but also culturally appropriate practices and with care not to exasperate already existing COVID-19 stigma. To this end, existing networks of community health workers and Red Cross and Red Crescent volunteers could be mobilised to liaise with community voluntary networks [31] to set-up local social care committees. These committees could be tasked with disseminating culturally appropriate information on behaviour change, facilitating a decision among the community as to which ‘green zone’ arrangement works for them, facilitating the community to contribute to the logistics of the effort, and coordinating the provision of food and supplies to high-risk residents. Such social mobilization and community engagement efforts could adapt relevant lessons learned and guidance developed in response to the Ebola epidemic [32], such as the need for locally-developed and facilitated action plans that are voluntary and protect basic human rights [33] and the satisfaction of basic non-COVID-19 needs. Local and international development and humanitarian actors, whose support accounts for a substantial (or, in the case of most camps, total) share of public service delivery, could contribute meaningfully by supplying infection control supplies (e.g. soap and water), supporting livelihoods, enabling local care committees and providing or strengthening mobile, dedicated medical treatment. This support would be mobilized through existing humanitarian coordination mechanisms and emergency operations centres.

### Infection control, active surveillance, and safeguarding

Stringent but realistic infection control and surveillance measures should accompany any of the options, as should some physical distancing within the green zone, especially under option 3. To facilitate acceptability, the green zone’s boundaries should probably remain virtual, but a single physical entry point, featuring handwashing facilities, should be established: food and other provisions should only be exchanged through this point. A meeting area where visitors can interact with loved ones at a safe distance and mobile, outpatient care can be provided could also be set up. Measures for active surveillance within the green zone, including appropriate screening and immediate isolation and where available, testing, of residents with symptoms consistent with COVID-19, should be added. This is in order to both provide early warning of infection and monitor the effects of shielding. Safeguarding mechanisms, such as providing additional support to individuals who are at risk of experiencing abuse or neglect, should also be introduced.

### When to start isolating? When to stand down?

Because of its short serial interval [34] and relatively high transmissibility, an uncontrolled COVID-19 epidemic would likely peak rapidly [35] depending upon various assumptions. While control measures currently being rolled out might slow this progression, the weakness of surveillance systems and inevitable implementation delays suggests a pragmatic need to roll out the proposed approach now.

However, isolating at-risk people has risks that should be acknowledged; if the virus infects the shielded group, it could move quickly among them, as noted in nursing homes in high-income countries. This will thus require effective surveillance and outbreak control in the green zone as well as outside. In separate guidance for camp and urban settings, we suggest mitigating interventions to reduce the risk of virus introduction and spread within green zones. Decisions about how to establish green zones must be weighed against continuing with the existing arrangements. Such decisions are difficult, and clearly ethically challenging. Isolating at-risk individuals for a long period of time will be psychologically taxing for the community, and as such, psychosocial support will be needed. Shielding should be discontinued as soon as safe to do so. In the absence of widespread testing, surveillance of adult mortality, the incidence of acute respiratory distress syndrome and/or continuous testing in sentinel sites, with simple stand-down thresholds (e.g. a period with no suspected cases of COVID-19 within a given radius) is preferable to relying on weaker national-level surveillance: this remains to be explored, as any syndromic approach is complicated by the high background of other respiratory infections.

## Conclusion

While the targeted approach we have outlined may only be one of several possible interventions, we believe that it may offer a realistic solution for allocating scarce resources to maximise impact in settings where scaling up treatment significantly is unlikely to be an option. Other feasible, high-yield interventions should be undertaken simultaneously, e.g. staying home if sick, limiting public transport use, reducing super-spreading events at funerals or other mass gatherings, promoting hand-washing, soap distribution and/or at least maintaining treatment coverage for risk-factor co-morbidities. Clearly, any shielding strategy would need to be predicated on sound, locally informed behavioural science and monitored for effectiveness, e.g. by measuring transmission or mortality within isolation ‘green zones’ and evaluating its potential under realistic modelling assumptions.

Whenever vaccines, improved therapeutics, or rapid testing for COVID-19 become available, these must be allocated equitably to low-income and crisis-affected populations. Until then, it is imperative that low-resource countries and humanitarian responses plan and roll out evidence-based, long-term strategies to mitigate their COVID-19 epidemics, starting now. Approaches such as containment of importation are likely to have exhausted their potential in the immediate future; not all interventions are of equal value, and the opportunity costs of emphasising one over the other should be considered. The price of inaction may be high. Sub-optimal, inefficient control interventions could, however, be just as costly.

## Data Availability

N/A

## References

[1] Gt Walker P, Whittaker C, Watson O, Baguelin M, Ainslie KEC, Bhatia S (2020). Report 12: the global impact of COVID-19 and strategies for mitigation and suppression.

[2] Truelove S, Abrahim O, Altare C, Lauer SA, Grantz KH, Azman AS (2020). The potential impact of COVID-19 in refugee camps in Bangladesh and beyond: a modeling study. Parmar P, editor. PLoS Med.

[3] House T, Keeling MJ (2009). Household structure and infectious disease transmission. Epidemiol Infect.

[4] Johnstone-Robertson SP, Mark D, Morrow C, Middelkoop K, Chiswell M, Aquino LDH (2011). Social mixing patterns within a south African township community: implications for respiratory disease transmission and control. Am J Epidemiol.

[5] le Polain de Waroux O, Cohuet S, Ndazima D, Kucharski AJ, Juan-Giner A, Flasche S, et al. Characteristics of human encounters and social mixing patterns relevant to infectious diseases spread by close contact: a survey in Southwest Uganda. BMC Infect Dis. 18(1):2018–172 [Cited 2020 Mar 18]. Available from: http://www.ncbi.nlm.nih.gov/pubmed/29642869. 10.1186/s12879-018-3073-1 PMC5896105 29642869

[6] Wong G, Liu W, Liu Y, Zhou B, Bi Y, Gao GF (2015). MERS, SARS, and Ebola: the role of super-spreaders in infectious disease. Cell Host Microbe.

[7] Kearney PM, Whelton M, Reynolds K, Muntner P, Whelton PK, He J (2005). Global burden of hypertension: analysis of worldwide data. Lancet.

[8] Zhou B, Bentham J, Di Cesare M, Bixby H, Danaei G, Cowan MJ (2017). Worldwide trends in blood pressure from 1975 to 2015: a pooled analysis of 1479 population-based measurement studies with 19·1 million participants. Lancet.

[9] Lancet Global Burden of Disease [Internet]. [cited 2020 Mar 18]. Available from: https://www.thelancet.com/gbd?source=post_page.

[10] Nordling L (2020). HIV and TB increase death risk from COVID-19, study finds—but not by much. Science (80-).

[11] Liu Y, Bi L, Chen Y, Wang Y, Fleming J, Yu Y, et al. Active or latent tuberculosis increases susceptibility to COVID-19 and disease severity. medRxiv. 2020; 2020.03.10.20033795.

[12] Ribacke KJB, Saulnier DD, Eriksson A, von Schreeb J (2016). Effects of the West Africa Ebola virus disease on health-care utilization - a systematic review. Front Public Health.

[13] Oppenheim B, Gallivan M, Madhav NK, Brown N, Serhiyenko V, Wolfe ND (2019). Assessing global preparedness for the next pandemic: development and application of an epidemic preparedness index. BMJ Glob Health.

[14] Ng Y, Li Z, Chua YX, Liang W, Zhao Z, Er B (2019). Morbidity and mortality weekly report evaluation of the effectiveness of surveillance and containment measures for the first 100 patients with COVID-19 in Singapore.

[15] Hellewell J, Abbott S, Gimma A, Bosse NI, Jarvis CI, Russell TW (2020). Feasibility of controlling COVID-19 outbreaks by isolation of cases and contacts. Lancet Glob Health.

[16] Response strategies for COVID-19 epidemics in African settings: a mathematical modelling study | CMMID Repository. [cited 2020 Apr 27]. Available from: https://cmmid.github.io/topics/covid19/covid-response-strategies-africa.html. 10.1186/s12916-020-01789-2 PMC7553800 33050951

[17] Parker B. The New Humanitarian | Yemen coronavirus lockdown to hamper relief effort. [Cited 2020 Mar 30]. Available from: https://www.thenewhumanitarian.org/news/2020/03/17/yemen-coronavirus-flights-lockdown.

[18] Graham H, Tosif S, Gray A, Qazi S, Campbell H, Peel D, et al. Administration d’oxygène aux enfants dans les hôpitaux: un examen réaliste, vol. 95: Bulletin of the World Health Organization. World Health Organization; 2017. p. 288–302. [Cited 2020 Jul 6]. Available from: http://www.who.int/bulletin/volumes/95/4/16-186676/en/.

[19] Gt Walker P, Whittaker C, Watson O, Baguelin M, Ainslie KEC, Bhatia S, et al. The Global Impact of COVID-19 and Strategies for Mitigation and Suppression. [cited 2020 Mar 30]. Available from: https://www.imperial.ac.uk/mrc-global-infectious-disease-analysis/covid-19/report-12-global-impact-covid-19/.

[20] Pung R, Chiew CJ, Young BE, Chin S, Chen MI-C, Clapham HE (2020). Investigation of three clusters of COVID-19 in Singapore: implications for surveillance and response measures. Lancet.

[21] Li R, Pei S, Chen B, Song Y, Zhang T, Yang W, et al. Substantial undocumented infection facilitates the rapid dissemination of novel coronavirus (SARS-CoV2). Science (80-). 2020;1:eabb3221. [Cited 2020 Mar 19]. Available from: https://www.sciencemag.org/lookup/doi/10.1126/science.abb3221. 10.1126/science.abb3221 PMC7164387 32179701

[22] Linton NM, Kobayashi T, Yang Y, Hayashi K, Akhmetzhanov AR, Jung S (2020). Incubation period and other epidemiological characteristics of 2019 novel coronavirus infections with right truncation: a statistical analysis of publicly available case data. J Clin Med.

[23] Liu Y (2020). The contribution of pre-symptomatic transmission to the COVID-19 outbreak | CMMID repository.

[24] Coronavirus lockdown in India: ‘Beaten and abused for doing my job’ - BBC News. [Cited 2020 Mar 30]. Available from: https://www.bbc.co.uk/news/world-asia-india-52063286.

[25] Moore D. “We fear, but have to work”: isolation not an option for the poor of Nairobi | Global development | The Guardian. [Cited 2020 Mar 30]. Available from: https://www.theguardian.com/global-development/2020/mar/27/we-fear-but-have-to-work-isolation-not-an-option-for-the-poor-of-nairobi-coronavirus.

[26] Sparks J. Coronavirus: The South African township where people just won’t follow the lockdown rules | World News | Sky News. [Cited 2020 Mar 30]. Available from: https://news.sky.com/story/coronavirus-the-south-african-township-where-people-just-wont-follow-the-lockdown-rules-11965027.

[27] Dong Y, Mo X, Hu Y, Qi X, Jiang F, Jiang Z, et al. Epidemiological characteristics of 2143 pediatric patients with 2019 coronavirus disease in China. Pediatrics. 2020; [Cited 2020 Mar 18]; Available from: https://pediatrics.aappublications.org/content/pediatrics/early/2020/03/16/peds.2020-0702.full.pdf.

[28] Handel A, Miller J, Ge Y, Fung IC-H (2020). If containment is not possible, how do we minimize mortality for COVID-19 and other emerging infectious disease outbreaks?. medRxiv.

[29] Wu Z, McGoogan JM (2020). Characteristics of and important lessons from the coronavirus disease 2019 (COVID-19) outbreak in China: summary of a report of 72 314 cases from the Chinese Center for Disease Control and Prevention. JAMA.

[30] Russell TW, Hellewell J, Jarvis CI, Van-Zandvoort K, Abbott S, Ratnayake R (2020). Estimating the infection and case fatality ratio for COVID-19 using age-adjusted data from the outbreak on the Diamond princess cruise ship. medRxiv.

[31] #عمل_الخير #السودان on Twitter: “#سوا_بنسلم هي مبادرة تستهدف دعم النساء العاملات باليومية في بيع الاطعمة والمشروبات في الشارع وذلك للحد من انتشار #فيروس_كورونا لمزيد من المعلومات والمساهمة ، الرجاء الاطلاع على البوسترات #سودان_بدون_كورونا https://t.co/u6pNyGteLz” / Twitter [Internet]. [cited 2020 Mar 30]. Available from: https://twitter.com/sharesudan/status/1243292945236652032.

[32] WHO | Community engagement and social mobilization. [Cited 2020 Mar 30]. Available from: https://www.who.int/csr/disease/ebola/training/community-engagement/en/.

[33] Skrip LA, Bedson J, Abramowitz S, Jalloh MB, Bah S, Jalloh MF (2020). Unmet needs and behaviour during the Ebola response in Sierra Leone: a retrospective, mixed-methods analysis of community feedback from the social mobilization action consortium. Lancet Planet Heal.

[34] Nishiura H, Linton NM, Akhmetzhanov AR (2020). Serial interval of novel coronavirus (COVID-19) infections. Int J Infect Dis.

[35] Kucharski AJ, Russell TW, Diamond C, Liu Y, Edmunds J, Funk S (2020). Early dynamics of transmission and control of COVID-19: a mathematical modelling study. Lancet Infect Dis.

